# Association Between the Use of a Mobile Health Strategy App and Biological Changes in Breast Cancer Survivors: Prospective Pre-Post Study

**DOI:** 10.2196/15062

**Published:** 2019-08-14

**Authors:** Mario Lozano-Lozano, Lucia Melguizo-Rodríguez, Carolina Fernández-Lao, Noelia Galiano-Castillo, Irene Cantarero-Villanueva, Lydia Martín-Martín, Manuel Arroyo-Morales

**Affiliations:** 1 Department of Physical Therapy, Faculty of Health Sciences University of Granada Granada Spain; 2 Sport and Health Joint University Institute Granada Spain; 3 Biohealth Research Institute Granada Spain; 4 “Cuidate” Support Unit for Oncology Patients Granada Spain; 5 Biomedical Group (BIO277), Department of Nursing Faculty of Health Sciences, University of Granada Granada Spain

**Keywords:** mHealth, interleukin-6, C-reactive protein, breast cancer survivors, low-grade inflammatory

## Abstract

**Background:**

There is a bidirectional relationship between chronic low-grade inflammation and cancer. Inflammatory markers, such as interleukin-6 (IL-6), have been associated with both the malignant transformation of epithelial cells and tumor progression, thus linking low-grade inflammation with a higher risk of cancer and recurrence in the survival phase. Therefore, they are considered valuable prognostic biomarkers. Knowing and finding appropriate primary prevention strategies to modify these parameters is a major challenge in reducing the risk of cancer recurrence and increasing survival. Different therapeutic strategies have shown efficacy in the modification of these and other biological parameters, but with contradictory results. There are apparently no strategies in which telemedicine, and specifically mobile health (mHealth), are used as a means to potentially cause biological changes.

**Objective:**

The objectives of this study were to: (1) check whether it is feasible to find changes in inflammation biomarkers through an mHealth strategy app as a delivery mechanism of an intervention to monitor energy balance; and (2) discover potential predictors of change of these markers in breast cancer survivors (BCSs).

**Methods:**

A prospective quasi-experimental pre-post study was conducted through an mHealth energy balance monitoring app with 73 BCSs, defined as stage I-IIIA of breast cancer and at least six months from the completion of the adjuvant therapy. Measurements included were biological salivary markers (IL-6 and C-reactive protein [CRP]), self-completed questionnaires (the European Organization for Research and Treatment of Cancer Quality of Life Questionnaire Core 30, the user version of the Mobile Application Rating Scale [uMARS] and an ad hoc clinical and sociodemographic questionnaire) and physical objective measures (accelerometry, weight and height). In addition, using the logging data of the mHealth app, the rate of use (in days) was recorded during the entire experimental phase of the study. Using Stata software, a paired two-tailed t test, Pearson and Spearman correlations, and a stepwise multiple regression analysis were used to interpret the data.

**Results:**

Analyzing changes in inflammatory biomarker concentrations after using the mHealth app, differences between preassessment CRP (4899.04 pg/ml; SD 1085.25) and IL-6 (87.15 pg/ml; SD 33.59) and postassessment CRP (4221.24 pg/ml; SD 911.55) and IL-6 (60.53 pg/ml; SD 36.31) showed a significant decrease in both markers, with a mean difference of –635.25 pg/ml (95% CI –935.65 to –334.85; P<.001) in CRP and –26.61 pg/ml (95% CI –42.51 to –10.71; P=.002) in IL-6. Stepwise regression analyses revealed that changes in global quality of life, as well as uMARS score and hormonal therapy, were possible predictors of change in CRP concentration after using the mHealth app. In the same way, the type of tumor removal surgery conducted, as well as changes in weight and pain score, were possible predictors of change in IL-6 concentration after using the app.

**Conclusions:**

In conclusion, through the results of this study, we hypothesize that there is a possible association between an mHealth energy balance monitoring strategy and biological changes in BCSs. These changes could be explained by different biopsychosocial parameters, such as the use of the application itself, quality of life, pain, type of tumor removal surgery, hormonal treatment or obesity.

## Introduction

There is a bidirectional relationship between chronic low-grade inflammation and breast cancer, as a tumor can produce an inflammatory environment and therefore a systemic immune response, but chronic inflammation can also both precede and promote the development of cancer [1]. There is even talk of considering inflammation to be an enabling feature of breast cancer, or the seventh hallmark of the disease along with the six hallmarks already identified by Hanahan and Weinberg [2,3]. Inflammation is a process, or bodily response, secondary to infection or sudden injury, and it is associated with the activation of various molecular mechanisms [4]. This response can be local or systemic, depending on the severity, and both indicate an imbalance of the metabolism of the affected tissues. This metabolic imbalance in the lesion is produced by an increase of immune cells as well as inflammatory parameters of great clinical importance, such as C-reactive protein (CRP) and its inducer interleukin-6 (IL-6) [4,5].

Once the inflammatory response ends, tissue metabolism is normalized. If this process of remission is interrupted by some circumstance, such as pathogens, toxins or other stimuli, healthy tissue could be damaged and produce what is known as persistent low-grade inflammation, or chronic inflammation [6]. It is the result of an immune system that overreacts so that the concentrations of inflammatory factors are higher than in a healthy population [5]. This systemic and chronic inflammation is widely associated with chronic diseases [6] and even symptomatology, as there is a positive association between increased levels of CRP and excess of adipose mass (excess weight and obesity), which is a factor that could be potentially modified with physical activity and diet [4,7]. Moreover, inflammatory markers such as IL-6 have also been associated with the malignant transformation of epithelial cells and tumor progression, associating low-grade inflammation with a higher risk of cancer and recurrence in the survival phase. Thus, these factors are considered valuable prognostic biomarkers in the population of those with cancer [1,8,9]. Therefore, knowing and finding appropriate primary prevention strategies to modify these parameters is a major challenge in the field, so as to reduce the risk of cancer recurrence and increase survival.

Different therapeutic strategies have shown efficacy in the modification of these and other biological parameters, but with contradictory results. The beneficial effects of physical exercise as a means of controlling low-grade inflammation have been amply demonstrated [10,11], even in breast cancer survivors (BCSs). A study conducted by Jones et al, in which they used a physical exercise program in BCSs, found a significant reduction of IL-6 [12]. Additional studies have evaluated other strategies, such as manual therapy [13], tai chi [14], mindfulness [15], or yoga [16], to reduce inflammation markers in different cancer populations. However, scientific evidence about strategies based on telemedicine are scarce, and they are practically nonexistent for cancer. A study conducted by Haggerty et al assessed two technology-based, 6-month, lifestyle interventions (telemedicine or text messaging) in obese women with endometrial hyperplasia, showing a reduction of some biomarkers such as IL-6 after the intervention [17]. Another study by Frank et al examined the effectiveness of telehealth coaching promoting nutrition and exercise in soldiers, evaluating biomarkers of bone health [18]. There are also some clinical trial protocols with no published results at present [19-21]. Therefore, at the moment there are no strategies in which telemedicine, and specifically mobile health (mHealth), are used as a delivery mechanism for interventions that could cause biological changes.

Low-grade inflammation is highly influenced by aspects such as obesity, fatigue or a sedentary lifestyle [22-24], and its relationship with chronic pathologies has been demonstrated. However, the issue of association, or the factors that influence its regulation through nonpharmacological and distance-based intervention strategies, remain unresolved [6]. In the biopsychosocial context that encompasses a subject with cancer, promoting changes through mHealth strategies in psychological, physical or social aspects is not entirely complicated (eg, quality of life) [25]. However, biological parameters have a high intersubject variability and are not usually addressed in these types of studies [24]. Therefore, understanding what factors can influence these parameters can help to develop mHealth-based strategies, thus increasing patient empowerment in regard to their health.

To the best of our knowledge, scientific evidence is scarce in regard to mHealth-based strategies related to tracking biomarkers of inflammation, and the importance of low-grade inflammation in cancer recurrence has already been demonstrated. Thus, the objectives of this study were to:

1. Check whether it is feasible to find changes in inflammation biomarkers through an mHealth strategy as a delivery mechanism of an intervention to monitor energy balance
2. Discover potential predictors of change of these markers in breast cancer survivors

## Methods

### Study Design, Participants, and Description of the Mobile Health App

A prospective quasi-experimental pre-post study was conducted through an mHealth app to monitor energy balance (BENECA mHealth app) with 73 BCSs, defined as stage I-IIIA of breast cancer and at least six months from the completion of adjuvant therapy (only hormonal therapy was allowed). Participants were recruited from the oncology units of San Cecili University Hospital and Virgen de las Nieves University Hospital, both in Granada, Spain, through their reference oncologists. All participants received oral and written information about the assessment protocols, mHealth app characteristics, and risks and benefits of the study, and then written consent was obtained from all of them. The Ethics Committee on Human Research (CEIH) from Granada province, Spain, approved this study (FIS, PI14-01627), which was performed in accordance with the Declaration of Helsinki [26]. The inclusion and exclusion criteria for this study are shown in Textbox 1.

After the initial assessment, all participants were invited to use the mHealth app for two months. In summary, the mHealth app was developed to help breast cancer survivors overcome energy balance challenges and aimed to both motivate and sensitize breast cancer survivors to adhere to fully personalized physical exercise programs and nutritional plans, in compliance with the international guidelines for cancer survivors. On first use, the users of the app recorded their personal and anthropometric data such as weight, height, age, and type of cancer. They were then asked to record what they ate (every item) and what they did (in terms of physical activity) the day before. Regarding food intake, BENECA uses a dietary record questionnaire structured with 6 consumption times. Regarding physical activity, patients could record the activities they completed during the day (intensity and duration) from 3 possible time periods (morning, afternoon, and evening). BENECA only records those activities that have a duration of at least 10 minutes.

Based on all this information, the mHealth app provided automatic feedback about a person’s energy balance or imbalance as well as nutritional information about what was ingested. In the presence of energy imbalance, it provided useful and simple tips to improve this imbalance. All these straightforward, daily notifications were based on the guidelines of the World Cancer Research Fund International [27], the strategies for physical activity and diet in patients with cancer from the American College of Sports Medicine [28], and the recommendations of the American Cancer Society [29,30]. The mHealth app was developed based on the theory of Learning, on Goal-Setting Theory, and on Social Cognitive Theory so as to include techniques such as reinforcement, facilitation, self-monitoring, goal setting, feedback on performance and reviewing goals, which have proven to be promising in increasing physical activity in different populations [31,32]. The technical characteristics of the mobile application [33], as well as validation of the energy balance monitoring system [34] and its feasibility [35], have been previously published.

Participants were able to contact a researcher at any time via WhatsApp, in case of technical problems or to discuss any doubts they had. In addition, an online video tutorial was available at any time.

**Inclusion and exclusion criteria.**

Inclusion criteria:

- Between 30 and 75 years of age.
- Body mass index >25 kg/m^2^.
- Stage I-IIIA of breast cancer.

Exclusion criteria:

- No medical clearance to participate.
- Any physical health condition that prevents them from walking.
- Any physical or mental health condition that prevents them from participating in assessments.
- No access to any mobile device or tablet with an internet connection.

### Outcomes Measures

To assess changes after use of the mHealth app, all measurements were taken at baseline and 8-weeks after having used it. Participants were called via phone for pre and postassessments and invited to Cuidate Support Unit for Oncology Patients, a clinical research center from the University of Granada, Spain. Measurements taken included biological markers, self-completed questionnaires, and both anthropometric and physical objective measures. In addition, using the logging data of the mHealth app, the rate of use (in days) was recorded during the entire experimental phase of the study.

### Biological Markers: Main Outcomes

Two salivary inflammatory markers were obtained: IL-6 and CRP. Salivary biomarkers have previously demonstrated the potential to be used for screening and research purposes [36].

#### Sample Handling and Preparation: Salivary Interleukin-6 and C-reactive Protein Concentrations

On the day of sample collection, the participants were informed of the requirements: they were not allowed to eat, drink or brush their teeth during the two hours prior to the collection, and they were not allowed to visit the dentist 24 hours before sampling, with the aim of reducing the risks of contamination. They were also not allowed to consume alcohol during the 12 hours prior to the collection of the sample, or to take acidic or high sugar foods. The saliva sample collection was done between 10:00 and 11:30 in the morning, and an attempt was made to match the time in the postassessment. Ten minutes before the collection of the sample, participants were asked to rinse their mouths with water. Saliva was collected by unstimulated passive drool for 3 minutes using a polypropylene vial. Participants were instructed to lean their heads forward, allowing the saliva to accumulate on the floor of the mouth. Immediately after collection, the sample was centrifuged at 3000-3500 rpm for 15 minutes (to remove mucins and other particles that might interfere with the results), and then the supernatant was stored in 200 μL tubes (total of 5 per participant). Finally, it was frozen and stored at –80°C for no longer than 3 months.

#### Sample Analysis (Enzyme Linked Immunosorbent Assay Procedures)

Once the sampling was completed, it was thawed completely until reaching room temperature prior to the completion of the solutions. The necessary sample was pipetted into dissolution tubes, and the residual saliva not analyzed was frozen again. The following enzyme linked immunosorbent assay (ELISA) kits were chosen: the Salimetrics C-Reactive Protein ELISA Kit (Kit number 1-3302, which is an enzyme-linked immunoassay specifically designed and validated for the quantitative measurement of salivary CRP), and the Salimetrics IL-6 ELISA Kit (Kit number 1-3602, which is a sandwich immunoassay specifically designed and validated for the quantitative measurement of salivary IL-6). Both have been designed and optimized for salivary research in humans. All analyses and calculations were performed following the manufacturer’s protocol, as described by Salimetrics. A total of 15 μL and 60 μL of saliva were required for the analyses of CRP and IL-6, respectively.

Once the reagents were prepared, we designed the plate where 100 μl of the samples were added, as well as the successive dilutions of the standard of each marker that would be used for the design of the standard curve. The sample was covered with an adhesive and incubated for two hours at room temperature before mixing at the mix plate at 500 rpm. Then, the plate was washed 4 times with wash buffer by filling and emptying the wells to remove the solution by either aspiration or plate inversion. After washing, antibody conjugate solution was added (100 μL/well) and then diluted in blocking buffer in a series of twofold dilutions. Then the plate was sealed and incubated for 2 hours at room temperature. After the incubation, we repeated the washing as described above. Once the wash was completed, the substrate solution was added, and the plate was incubated in the dark at room temperature for 30 minutes before then mixing for 5 minutes on a plate rotator at 500 rpm. Then, we stopped the reaction by adding the stop solution (50 μl). The solution was mixed at the plate rotator for 3 minutes at 500 rpm. The absorbance was then measured with a spectrophotometer (Biotek ELx800) at 450 nm, according to kit manufacturers. Results were compared with a standard curve that was previously designed. All standards, controls and samples were analyzed in duplicate.

### Self-Completed Questionnaires

The European Organization for Research and Treatment of Cancer Quality of Life Questionnaire Core 30 (EORT QLQ-C30) version 3.0 was used to measure quality of life of the participants. It is a questionnaire specifically designed to evaluate general aspects of quality of life of patients with cancer. It is composed of a global scale of health status, five functional scales (in which the higher the score, the higher the quality of life reported) and eight symptom scales (in which the higher the score, the greater the symptoms reported). This instrument has shown adequate reliability [37,38].

The user version of the Mobile Application Rating Scale (uMARS) was used to measure the satisfaction and quality of usage of the mHealth app. This questionnaire is composed of 23 elements grouped into different sections, each of them evaluated independently through a Likert scale of 1 to 5 points (5 being excellent). Finally, the average score is calculated. This scale has been validated and has proven to be simple, objective and reliable [39].

An ad hoc questionnaire was used to collect clinical and sociodemographic characteristics of participants, including the stage of breast cancer, the type of tumor removal surgery, and the medical treatment and hormonal therapy. The stage of breast cancer could be I, II or III-A, the type of surgery was categorized in increasing order according to invasion of the surgery method (lumpectomy, quadrantectomy, unilateral mastectomy and bilateral mastectomy), the medical treatment was either a neoadjuvant or adjuvant treatment, and the hormonal therapy was registered as either taking or not taking hormonal treatment, as well as its typology.

### Anthropometric and Physical Objective Measures

A preprogrammed triaxial accelerometer (ActiGraph GT3X+, Pensacola, Florida) was used to collect data on participants' physical activity over 8 consecutive days, together with a questionnaire diary based on a previously published protocol of use and analysis [40,41]. Only the records of more than 4 days, and of at least 10 hours per day, were included in the analysis. Minutes of vigorous-to-moderate physical activity (MVPA) were recorded.

Weight (kg) and height (cm) were measured with light clothing and without shoes. Weight was measured using an electronic scale (model SECA 869, Hamburg, Germany), and height was measured in the Frankfort plane using a stadiometer (model SECA 213).

### Statistical Analysis

Measures of central tendency and dispersion were used for continuous variables with a normal distribution. Categorical variables were reported as proportions (%). The Kolmogorov-Smirnov test was used to check the normal distribution of the data. To evaluate the differences in the biological variables (CRP and IL-6) between baseline and after 8 weeks of use of the app, a paired two-tailed *t* test was used. To analyze the correlation between the different variables, Pearson and Spearman correlation were applied as appropriate. In this correlation analysis, the change variable (difference between postassessment and preassessment) was used with quantitative variables: biological variables, quality of life (EORT QLQ-C30 global score, fatigue and pain), satisfaction with the app (uMARS global score), use of the app (in days), MVPA (accelerometry) and weight. The change variable was also used to measure changes in clinical variables such as type of tumor removal surgery, stage of breast cancer, medical treatment and hormonal therapy. Dispersion diagrams were used to study the assumptions of normality, linearity and homoscedasticity. To determine which variables could explain the variation in CRP and IL-6 concentrations, a stepwise multiple regression analysis was used. For the regression model with the dependent variable of CRP, the score changes in general quality of life, hormonal treatment, quality and satisfaction were considered independent variables.

For the regression model with the dependent variable IL-6, type of tumor removal surgery, and score changes in both perceived pain and weight were considered independent variables. To be included in the multiple regression analysis, the independent variables had to have a correlation coefficient of *r*>0.20 between the dependent variable and the independent variable, and they had to be significant [42]. The possible collinearity between the independent variables was studied, and then the final model was validated using bootstrapping (the start-up method was carried out with repeated samples of the same size to replace the original samples). Two thousand repetitions were produced to estimate the confidence intervals accelerated and corrected for the starting bias. For statistical analyses, the level of significance was set at *P*<.10. All analyses were performed using the software Stata version 14 (Statacorp, College Station, Texas). At least two experiments were performed in all assays.

## Results

### User Statistics and Clinical Characteristics

Participants were, on average, 51.35 (SD 8.58) years of age, with a body mass index (BMI) of 28.86 (SD 8.58). A total of 64% of the BCSs listed their civil status as married and 21% as single, with 41% having an educational status of higher education and 31% having unfinished studies or primary school. Table 1 summarizes clinical and sociodemographic participants’ characteristics.

Participants showed moderate quality of satisfaction (score range=0-5) with the mHealth app (mean 3.71 points; SD 0.47 points), high app usage (mean 47.9 days; SD 10.40; max=56 days), and moderate to low scores (range 0-100) in general quality of life (mean 57.6; SD 14.07), fatigue (mean 23.14; SD 15.46), and pain (mean 45.66; SD 25.91). Finally, mean weight was 72.56 kg (SD 10.85 kg) and the mean MVPA was 47.27 (SD 23.41). Analyzing changes in inflammatory biomarker concentrations after using the mHealth app, differences between preassessment CRP (4899.04 pg/ml; SD 1085.25) and IL-6 (87.15 pg/ml; SD 33.59) and postassessment CRP (4221.24 pg/ml; SD 911.55) and IL-6 (60.53 pg/ml; SD 36.31) showed a significant decrease in both of them, with a mean difference of –635.25 pg/ml (95% CI –935.65 to –334.85; *P*<.001) in CRP and –26.61 pg/ml (95% CI; –42.51 to –10.71; *P*=.002) in IL-6.

**Table 1:** Participants’ demographics (N=73).

Variables		Participants
Age (years), mean (SD)		51.35 (8.58)
**Marital status, n (%)**		
	Single	15 (20.6)
	Married	47 (64.4)
	Divorced	7 (9.6)
	Other	4 (5.5)
**Education, n (%)**		
	No education	1 (1.4)
	Primary studies	22 (30.1)
	Secondary studies	20 (27.4)
	Higher education	30 (41.1)
**Employment, n (%)**		
	Housewife	18 (24.7)
	Employee	28 (38.4)
	Low	10 (13.7)
	Unemployed by the disease	17 (23.3)
**Cancer stage, n (%)**		
	I	8 (11.3)
	II	37 (52.1)
	IIIA	26 (36.6)
**Surgery, n (%)**		
	Lumpectomy	24 (32.8)
	Quadrantectomy	12 (16.4)
	Unilateral mastectomy	26 (35.6)
	Bilateral mastectomy	11 (15.1)
**Medical treatment, n (%)**		
	None	5 (6.9)
	Radiation therapy alone	6 (8.2)
	Chemotherapy alone	5 (6.9)
	Chemotherapy and radiation therapy	48 (65.8)
	Adjuvant chemotherapy	6 (8.2)
	Neoadjuvant chemotherapy	3 (4.1)

### Correlation Analyses

Significant negative correlations were found between changes in CRP concentration and EORT QLQ C30 general quality of life (*r*=–0.281; *P*=.03), with hormonal therapy (*r*=–0.235; *P*=.07), with uMARS score (*r*=–0.284; *P*=.02) and with mHealth app usage (*r*=–0.263; *P*=.04). In addition, significant positive correlations were found between change in IL-6 concentration and EORT QLQ C30 pain (*r*=0.404; *P*=.01), with weight (*r*=0.301; *P*=.06) and with type of tumor removal surgery (*r*=0.311; *P*=.05).

In addition, significant correlations existed among the independent variables (Table 2) but was only high between uMARS score and mHealth usage (*r*=0.907; *P*<.001). Therefore, considering multicollinearity possible (defined as *r*>0.70), only uMARS score was included in the regression analyses.

**Table 2:** Pearson product-moment correlation matrix for study variables.

Variable		Δ^a^CRP^b^		ΔIL-6^c^		ΔC30^d^ QoL^e^		ΔC30 Fatigue		ΔC30 Pain		ΔWeight		ΔMVPA^f^		Age		Stage BC^g^		Surgery type		Hormonal therapy		uMARS^h^		mHealth^i^ use	
ΔCRP		1.00		—^j^		—		—		—		—		—		—		—		—		—		—		—	
**ΔIL-6**		0.191		1.00		—		—		—		—		—		—		—		—		—		—		—	
	*P* value		.28		—		—		—		—		—		—		—		—		—		—		—		—
**ΔC30 QoL**		–0.281		–0.168		1.00		—		—		—		—		—		—		—		—		—		—	
	*P* value		.03		.30		—		—		—		—		—		—		—		—		—		—		—
**ΔC30 fatigue**		0.054		0.208		–0.527		1.00		—		—		—		—		—		—		—		—		—	
	*P* value		.68		.20		<.001		—		—		—		—		—		—		—		—		—		—
**ΔC30 pain**		0.153		0.404		–0.35		0.678		1.00		—		—		—		—		—		—		—		—	
	*P* value		.24		.01		.002		<.001		—		—		—		—		—		—		—		—		—
**ΔWeight**		0.088		0.301		–0.183		0.088		0.04		1.00		—		—		—		—		—		—		—	
	*P* value		.50		.06		.12		.46		.73		—		—		—		—		—		—		—		—
**ΔMVPA**		0.099		0.011		–0.023		0.135		0.187		–0.116		1.00		—		—		—		—		—		—	
	*P* value		.47		.95		.85		.29		.14		.36		—		—		—		—		—		—		—
**Age**		–0.139		–0.04		0.259		–0.314		–0.301		0.20		–0.304		1.00		—		—		—		—		—	
	*P* value		.28		.8		.03		.01		.01		.09		.01		—		—		—		—		—		—
**Stage BC**		0.075		0.143		–0.055		0.079		–0.083		–0.045		–0.101		0.143		1.00		—		—		—		—	
	*P* value		.57		.38		.65		.51		.49		.71		.43		.23		—		—		—		—		—
**Surgery type**		–0.158		0.311		0.092		0.023		0.009		–0.023		–0.191		0.110		0.282		1.00		—		—		—	
	*P* value		.22		.05		.44		.84		.93		.85		.13		.35		.02		—		—		—		—
**Hormonal therapy**		–0.235		–0.001		0.142		–0.161		–0.161		0.088		0.018		0.285		0.052		0.247		1.00		—		—	
	*P* value		.07		>.99		.24		.18		.18		.46		.88		.02		.66		.04		—		—		—
**uMARS**		–0.284		–0.086		–0.105		0.09		0.188		–0.028		0.101		–0.309		–0.167		0.147		0.049		1.00		—	
	*P* value		.02		.57		.38		.45		.11		.81		.42		.01		.16		.23		.68		—		—
**mHealth use**		–0.263		–0.127		–0.101		0.112		0.184		–0.026		0.081		–0.402		–0.122		0.144		0.014		0.907		1.00	
	*P* value		.04		.43		.40		.35		.12		.82		.52		<.001		.31		.22		.91		<.001		—

^a^Δ: change between postassessment and preassessment.

^b^CRP: C-reactive protein.

^c^IL-6: interleukin-6.

^d^C30: EORT QLQ C-30 questionnaire.

^e^QoL: quality of life.

^f^MVPA: minutes of vigorous-to-moderate physical activity.

^g^BC: breast cancer.

^h^uMARS: user version of the Mobile Application Rating Scale.

^i^mHealth: mobile health.

^j^Not applicable.

### Regression Analyses

Stepwise regression analyses revealed that changes in global quality of life, as well as uMARS score and hormonal therapy, were possible predictors of change in CRP concentration after using the app (Table 3). In the same way, the type of tumor removal surgery, as well as changes in weight and pain score, were possible predictors of change in IL-6 concentration after using the app (Table 4). For both tables, *r*^2^ denotes the variability of change in biomarker concentration explained by the predictors in percent.

**Table 3:** Summary of stepwise regression analyses to determine predictors of change in C-reactive protein concentration (*r*^2^=19%).

Independent variables	Unstandardized coefficients, β^a^	95% CI for β	Bootstrap BCA^b^, 95% CI	Bootstrap, β	Standardized coefficients, β	*t*	*P* value
Interceptil	2496.949	218.504-4775.395	4.641-4989.268	2496.949	—^c^	2.2	.03
Hormonal therapy	–110.304	–286.447 to 65.839	–272.774 to 52.166	–110.304	–0.155	–1.25	.22
uMARS^d^ score	–728.786	–1338.675 to –118.898	–1396.04 to –61.533	–728.785	–0.289	–2.39	.02
Δ^e^Global QoL^f^	–18.601	–36.253 to –0.945	–37.022 to –0.177	–18.605	–0.261	–2.11	.04

^a^β: regression coefficient.

^b^BCA: bias-corrected and accelerated.

^c^Not applicable.

^d^uMARS: user version of the mobile application rating scale.

^e^Δ: change between postassessment and preassessment.

^f^QoL: quality of life.

**Table 4:** Summary of stepwise regression analyses to determine predictors of change in IL-6 concentration (*r*^2^=26%).

Independent variables	Unstandardized coefficients, β^a^	95% CI for β	Bootstrap BCA^b^, 95% CI	Bootstrap, β	Standardized coefficients, β	*t*	*P* value
Interceptil	–36.498	–68.898; –4.098	–71.773; –1.223	–36.498	—^c^	–2.28	.03
Type of surgery	8.219	–4.604; 21.04	–5.383; 21.821	8.22	0.194	1.3	.20
Δ^d^Weight	4.456	–1.263; 10.176	–0.582; 9.495	4.455	0.23	1.58	.08
ΔPain score	0.667	0.054; 1.279	0.003; 1.33	0.667	0.328	2.21	.03

^a^β: regression coefficient.

^b^BCA: bias-corrected and accelerated.

^c^Not applicable.

^d^Δ: change between postassessment and preassessment.

## Discussion

The objective of this study was to determine the preliminary results of the possible association between the use of an mHealth strategy app as a delivery mechanism to monitor energy balance in cancer and the reduction of systemic inflammation markers, as well as to suggest possible predictors of this change. Current findings suggest that after two months of use of the app, a significant reduction of these markers can be observed. Thus, there could be a possible association between the two. In addition, the change in weight, pain and quality of life, as well as the type of tumor removal surgery, hormone therapy and the uMARS score, can have a contribution in the changes found in the concentrations of CRP and IL-6.

A system of monitoring energy balance through an mHealth app seems to reduce the biological parameters of systemic inflammation (CRP and IL-6). Our results suggest that after two months of use of the mHealth app, based on the monitoring of energy balance (in terms of diet and physical activity), the concentration of CRP and IL-6 are significantly reduced in BCSs. In fact, this change has a moderate effect size in CRP (Cohen *d*=–0.640; 95% CI –0.985 to –0.293) and a high effect size in IL-6 (Cohen *d*=–0.805; 95% CI –1.225 to –0.379). A study by Skogstad et al was the only one found with a design similar to ours, as it used a virtual internet physical activity motivation strategy in which some biological parameters were measured [43]. In this study, participants were included in a motivational physical activity program in which they measured their steps using a wrist-band accelerometer. However, unlike our study, no differences were found in CRP concentration after the intervention, perhaps because their study target population were healthy workers without pathology. The effect size reported for both our biomarkers supports the hypothesis that these changes are not due to time, but it is important to remark that the quasi-experimental pre-post design of our study does not allow us to affirm that the changes found are only attributable to the use of the app. Therefore, a controlled and randomized clinical trial should be carried out in the future.

Biological parameters of systemic inflammation can be mediated or modified by lifestyles changes such as physical activity and diet [4,6,7]. This study is the first to examine clinical and anthropometric factors that affect changes in these biological parameters, after using a mobile strategy to monitor energy balance in breast cancer survivors. Because rehabilitation strategies focus on face-to-face or distance physical activity and diet programs, understanding the potential determinants of reducing inflammation markers can help design more effective intervention strategies.

The results of our study show that possible moderators of a reduction in CRP concentration include not receiving hormonal therapy, as well as having higher satisfaction and changes in quality of life (the higher quality of life change, the lower the CRP concentration). The role of estrogen in inflammation is poorly understood, the mechanism is not well studied, and its relationship is very complex [44], and different studies show contradictory results depending on different pathologies, with some showing they are associated with an inflammatory activity, while others show a proinflammatory role [44]. The differences found in pre and postmenopausal women suggest that the peripheral production of estrogens plays an important role in these differences [44-47]. Our results provide new evidence in this regard, since not having received hormone therapy may be a predictor of a greater reduction in CRP concentration in female survivors of breast cancer. However, estrogen’s relationship with quality of life has been considered from another point of view. We understand that it is not that a higher perception of quality of life is a predictor of a reduction of proinflammatory markers but rather the other way around, that the diminished inflammatory state is associated with an increase in the quality of life [48,49]. Ultimately, our results suggest a higher score in uMARS as a predictor of the change in CRP concentration. In addition, there is a strong association between satisfaction and quality with the amount of time spent on the mHealth app. If women with the highest score in uMARS use the mHealth app more, then the reduction in inflammatory markers could be due to the direct relationship caused by a healthier lifestyle [6,50,51].

The results of our study also show possible moderators of the reduction in IL-6 include the type of tumor removal surgery (less invasive surgery), as well as changes in both weight and pain (the greater the reduction of these factors, the greater the reduction of IL-6). These results are consistent with the known bidirectional relationship between obesity and low-grade inflammation, which contributes to systemic metabolic dysfunction that is associated with obesity-linked disorders [4]. In the same way, an inflammatory reaction is also mediated by the classic cardinal signs of inflammation (eg, pain) [52]. Therefore, it is logical to think that a reduction in pain reported by breast cancer survivors can be a predictor of a reduction in IL-6 concentration such as that observed in our results. Finally, there is a lot of scientific evidence to support the use of minimally invasive surgical techniques since they don’t raise inflammatory reactants as much, and these findings may support the relationship between IL-6 and the type of tumor removal surgery found in our results [53-56].

It is worth highlighting some strengths and limitations of the present study. The main strength lies in the nature of the study. To the best of our knowledge, this is the first study that proposes a mobile strategy to monitor energy balance as a mediator in the reduction of proinflammatory markers in BCSs. If future research supports our results, we will have found another support strategy for cancer survivors that is low cost and accessible to everyone and which could reduce markers highly related to the risk of recurrence. However, there are also many limitations to be noted. The main limitation lies in the design of the study, as well as the sample size, which prevents us from speaking in terms of causality and effectiveness. In addition, the *r*^2^ obtained in the multiple regression models was low. However, we must not forget that we are trying to explain biological parameters with nonbiological variables. Our results may support the biopsychosocial model, since it shows how biology can be modified through these variables. Other biological parameters that can justify the rest of the variability that has remained to be explained should be taken into account in the future.

In conclusion, through the results of this study, we hypothesize that there is a possible association between an mHealth energy balance monitoring strategy app and biological changes in BCSs. These changes could be explained by different biopsychosocial parameters such as the use of the application itself, quality of life, pain, type of tumor removal surgery, hormonal treatment or obesity. Future studies should be carried out with a specific focus on all these biological parameters, and with an appropriate study design, to support these findings.

## Abbreviations

BCS: breast cancer survivor
BMI: body mass index
CEIH: Ethics Committee on Human Research
CRP: C-reactive protein
ELISA: enzyme linked immunosorbent assay
EORT QLQ-C30: the European Organization for Research and Treatment of Cancer Quality of Life Questionnaire Core 30
IL-6: interleukin-6
MVPA: minutes of vigorous-to-moderate physical activity
RCT: randomized controlled trial
uMARS: user version of the mobile application rating scale
WCRF: World Cancer Research Fund International
